# A hybrid mamba-transformer architecture fusing clinical and genetic features for gestational diabetes mellitus prediction

**DOI:** 10.1371/journal.pone.0355729

**Published:** 2026-08-12

**Authors:** Ji Huang, Wenbing Shi, Lan Lin, Zhongliang Wei, Qianwen Li

**Affiliations:** 1 Information Management Center, Fuzhou University Affiliated Provincial Hospital, Fuzhou, Fujian, China; 2 School of Computer Science and Engineering, Anhui University of Science and Technology, Huainan, Anhui, China; 3 Fujian Provincial Key Laboratory of Medical Big Data Engineering, Fuzhou University Affiliated Provincial Hospital, Fuzhou, Fujian, China; University of Essex Faculty of Science and Engineering, UNITED KINGDOM OF GREAT BRITAIN AND NORTHERN IRELAND

## Abstract

Gestational diabetes mellitus (GDM) is a common disorder of glucose metabolism during pregnancy. Early GDM prediction is crucial for reducing adverse maternal and neonatal outcomes. This paper proposes a hybrid Mamba-Transformer architecture that aggregates clinical and genetic features for GDM prediction. First, a correlation-driven weighted fusion method for clinical and genetic features is introduced. The integrated representation not only enhances feature representation but also highlights the interactive relationship between genetic susceptibility and clinical factors. Second, a sliding window approach is applied to reconstruct the sample sequences from the preprocessed data, generating augmented instances as model input. This transforms isolated individual features into context-aware group features, enabling the effective capture of both population-level heterogeneity and individual risk. Finally, the hybrid Mamba-Transformer architecture is constructed and trained on the publicly available competition dataset (DMRPD) from the Alibaba Cloud Tianchi platform. The model employs a modular and extensible encoder-decoder structure, where the Mamba module serves as an efficient feature extractor for dependencies, while the Transformer module performs deep semantic modeling and sequence abstraction. Experimental results indicate that the proposed method achieves competitive performance compared with other representative models. Specifically, the model attained an AUC of 0.825 on the test set, with sensitivity and specificity at the optimal threshold (0.526) of 0.827 and 0.729, respectively, suggesting reliable discriminative performance on the test set. These findings suggest that the proposed method may provide a useful approach for early GDM risk prediction and could potentially support more targeted screening strategies. However, further validation using larger and independent cohorts is required to confirm its generalizability and clinical applicability.

## 1 Introduction

Gestational diabetes mellitus (GDM) is a glucose metabolism disorder of varying degrees that occurs during pregnancy. It is a glucose tolerance disorder that first appears or is diagnosed during pregnancy. The main feature of GDM is that pregnant women have glucose metabolism disorders during pregnancy, but their blood sugar levels have not yet reached the diagnostic criteria for overt diabetes [1, 2]. GDM not only affects the health of pregnant women but may also lead to a series of complications, such as fetal malformation, macrosomia, and premature birth, increasing perinatal risks for mothers and infants. In addition, the probability of GDM patients developing type 2 diabetes mellitus (T2DM) after delivery is significantly increased [3–5]. Therefore, early GDM prediction has significant clinical value. It helps to develop personalized intervention measures to reduce the occurrence of pregnancy complications.

In the GDM prediction, many studies at home and abroad have focused on the relationship between specific indices and GDM. For example, some studies have shown that factors such as the atherogenic index of plasma, gastrointestinal microbiome, and obesity are associated with GDM [6, 7]. The epidemiological study of GDM mainly relies on epidemiological models and experimental data. It evaluates the GDM risk by analyzing factors such as population characteristics, geographical differences, and environmental pollution [8, 9]. However, these methods require more prospective studies and high-quality in vivo and in vitro experiments to investigate their specific effects and mechanisms, and it is challenging to consider complex genetic interactions.

In recent years, with the advancement of genomics and bioinformatics technologies, researchers have begun to focus on the role of genetic factors in the occurrence of GDM. Gene polymorphism studies have shown that certain specific gene mutations or variations are closely related to the occurrence of GDM. For example, recent studies have found that rs62069863 in the TRPV3 gene, rs2232016 in the PRMT6 gene, and rs10460009 in the LPIN2 gene are new genetic susceptibility loci for GDM in the Chinese Han population [10]. However, the impact of a single gene is limited. How to effectively integrate routine physical examination information and multi-gene information for intelligent GDM prediction is still a hot topic in current research.

GDM can cause adverse consequences for mothers and their newborns. Pregnant women in some low and middle-income regions or countries often cannot receive early clinical intervention due to limited medical resources. Machine learning methods have good application prospects in the diagnosis of GDM. Algorithms such as SVM, Random Forest, AdaBoost, Decision Tree, XGBoost, and GBDT have been used to build GDM prediction models [11, 12]. These methods can automatically learn complex patterns in data, but they still face problems such as high-dimensional feature selection and low generalization ability.

With the development of deep learning, researchers have begun to use deep neural networks for GDM prediction. For example, patients at high risk of GDM are identified based on recurrent neural networks and Bayesian optimization [13]. It provides auxiliary decision support for clinicians and reduces unnecessary oral glucose tolerance tests. In addition, the Transformer architecture has also been applied in the fields of bioinformatics and medical prediction due to its outstanding performance in natural language processing tasks [14, 15]. As a new sequence modeling method, Mamba has attracted widespread attention from researchers in the field of bioinformatics due to its efficient long sequence processing capabilities and computational efficiency [16]. The Mamba model can effectively model long-range dependencies. It has advantages over the traditional Transformer model in terms of computational complexity and training efficiency.

Although these methods have made some research progress in their focused areas, there are still some limitations. Traditional statistical methods and machine learning methods have limited predictive ability when dealing with high-dimensional, non-linear, and complex clinical and genetic interactions. Deep learning is often considered a black box model. Its feature selection is opaque, or the algorithm lacks clinical logic, making it difficult for clinicians to understand its prediction basis. This has affected its trust and adoption in clinical practice. The traditional Transformer structure has a high computational complexity when processing long sequence data. Its attention mechanism may not be able to effectively capture long-distance dependencies, resulting in information loss or increased noise. Therefore, constructing efficient predictive models capable of capturing the intricate interactions between clinical and genetic features holds significant theoretical importance and practical value.

In response to the above challenges, this paper proposes a hybrid Mamba-Transformer architecture fusing clinical and genetic features for GDM prediction. The proposed architecture is designed to capture the complex interactions between heterogeneous features while improving the modeling of long-range dependencies. By integrating genetic and clinical features, this method aims to capture complex feature interactions and improve GDM risk prediction. The effectiveness of this method is evaluated through experiments on a real-world GDM dataset. The main innovations and contributions of this study are summarized as follows:

(1) We introduce a correlation-driven weighted fusion method for genetic and clinical features. This method enhances feature representation and emphasizes the interactive relationship between genetic susceptibility and clinical factors. In addition, it overcomes the limitation of existing GDM prediction models that often neglect genetic contributions.
(2) We construct a hybrid Mamba-Transformer architecture for GDM prediction. By integrating the complementary strengths of Mamba and Transformer, the proposed framework enhances the modeling of complex dependencies and improves the representation of heterogeneous clinical and genetic information.
(3) In architectural design, we develop a flexible and scalable model structure that can adapt to different application scenarios and varying task complexities, facilitating transferability and applicability across diverse prediction scenarios.

The rest of this paper is organized as follows: Section 2 provides a brief review of related works on GDM prediction. Section 3 presents the overall architecture and detailed design of the proposed method. Section 4 describes the experimental procedures and results analysis. Finally, a discussion and conclusion are made in Sections 5 and 6.

## 2 Related works

In recent years, various approaches have been proposed to analyze and predict the risk of GDM, including statistical and epidemiological analyses, machine learning techniques, and deep learning models. In this section, we review recent advances related to GDM risk analysis and prediction.

### 2.1 Statistical and epidemiological approaches

Statistical and epidemiological approaches have been widely used to identify GDM-related risk factors, investigate associations among clinical and biological variables, and develop risk prediction models based on population-level medical data. These approaches have provided valuable insights into the associations between clinical characteristics, biological markers, environmental exposures, and GDM risk. For example, Man et al. [17] developed a diabetes risk prediction model using Cox proportional hazards regression based on baseline clinical variables from women with prediabetes and prior GDM. In addition to clinical indicators, observational studies have explored potential biological mechanisms underlying GDM. Hong et al. [18] investigated plasma amino acid profiles, while Faurø et al. [19] examined lipid-related biomarkers associated with GDM risk.

Recent epidemiological studies have further expanded the understanding of GDM by incorporating environmental and lifestyle-related factors. He et al. [20] analyzed the association between residential green exposure and glycemic levels, while Mao et al. [21] and Li et al. [22] investigated the effects of chemical exposure and air pollution on GDM incidence. In predictive modeling, Manna et al. [23] integrated clinical biomarkers with metabolomics information to construct a multivariate prediction model, while Lyu et al. [24] demonstrated that simplified models based on routinely available clinical features could achieve practical risk prediction. Furthermore, psychosocial factors have also been considered in GDM assessment, as demonstrated by Kumar et al. [25]. Although statistical and epidemiological approaches remain valuable for identifying risk factors, their ability to capture complex nonlinear interactions among heterogeneous variables is limited.

### 2.2 Enhanced machine learning approaches for risk prediction

To overcome the limitations of conventional statistical models, enhanced machine learning approaches have increasingly been applied to GDM and diabetes-related prediction tasks. These approaches incorporate ensemble strategies, feature engineering, optimization techniques, and interpretability methods, enabling them to model nonlinear relationships and complex feature interactions. Among these methods, ensemble learning algorithms, particularly gradient boosting models, have shown promising predictive performance. Kumar et al. [26] combined CatBoost with Shapley feature attribution to develop an interpretable GDM prediction model, while Belsti et al. [27] demonstrated the effectiveness of CatBoost compared with traditional statistical methods using routine prenatal data.

Beyond model selection, recent studies have highlighted the importance of feature engineering, optimization strategies, and data quality enhancement. Olisah et al. [28] explored feature selection and missing value processing strategies, while Karuppasamy et al. [29] proposed a hybrid optimization-based stacking framework. Zhao et al. [30] and Wang et al. [31] further investigated ensemble learning and feature selection techniques to improve prediction performance. In addition, Xu et al. [32] focused on improving data reliability through label noise filtering, and Ramsingh et al. [33] explored a scalable Hadoop-based framework for analyzing large-scale social media data related to diabetes. Despite these advances, machine learning models generally depend on manually designed features and may have limited capability in automatically learning high-level representations from heterogeneous clinical and genetic information.

### 2.3 Deep learning approaches in medical prediction

Deep learning approaches have recently attracted increasing attention in medical prediction because of their ability to automatically extract complex representations from high-dimensional data. Zheng et al. [34] evaluated multiple deep learning architectures for GDM prediction and reported the effectiveness of attention-based networks. Sumathi et al. [35] developed a deep-stacked autoencoder framework for automated GDM diagnosis. Other studies have focused on improving robustness and interpretability. Shaheen et al. [36] addressed class imbalance through sampling strategies, while Dhal et al. [37] optimized deep neural networks using hybrid optimization techniques. Singh et al. [38] and Cheng et al. [39] further incorporated explainability and semantic enhancement strategies to improve model transparency and representation capability.

Recently, Transformer-based architectures have emerged as powerful approaches for medical prediction due to their ability to capture long-range dependencies through self-attention mechanisms. Oulhadj et al. [40] applied a vision transformer-based framework for diabetic retinopathy prediction and achieved competitive performance across multiple datasets. However, the quadratic computational complexity of self-attention limits the scalability of Transformers for long sequences or large-scale applications. To address this limitation, Mamba, a selective state space model, has recently been introduced as an efficient alternative for sequence modeling. Nataliani et al. [41] incorporated Mamba into a mobile U-Net framework for diabetic foot ulcer segmentation, demonstrating its potential to achieve a balance between computational efficiency and predictive performance.

Overall, existing studies have significantly advanced GDM prediction through statistical analysis, machine learning, and deep learning techniques. However, several challenges remain. Statistical approaches are limited in modeling nonlinear feature interactions, while conventional machine learning methods often rely on handcrafted feature engineering. Although deep learning methods provide stronger representation capabilities, existing studies have rarely investigated the effective integration of heterogeneous clinical and genetic information for GDM prediction. Moreover, the potential of combining Mamba with Transformers remains underexplored in this field. Therefore, this study proposes a hybrid Mamba-Transformer framework that integrates clinical indicators and genetic features for GDM risk prediction, aiming to exploit complementary information from heterogeneous data sources and improve predictive performance.

## 3 Methods

This section details the proposed methods of GDM prediction, which will be elaborated from two aspects: feature fusion and sample reconstruction, and the construction of the MT-GDM model.

### 3.1 Feature fusion and sample reconstruction

**Fig 1:**
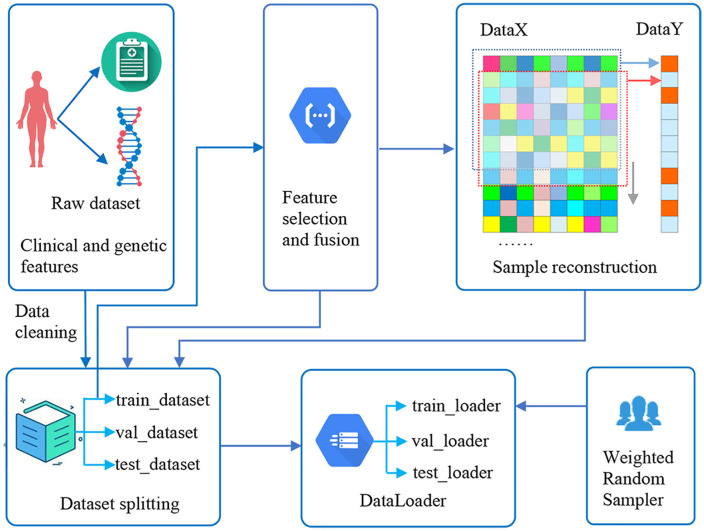
The process of data preprocessing, feature fusion, and sample reconstruction.

The data source used in this study is the Diabetes Mellitus Risk Prediction Dataset (DMRPD), a publicly available dataset released through the Alibaba Cloud Tianchi Precision Medicine Competition [42]. Clinical features, such as age, body mass index (BMI), blood pressure, blood glucose levels, and lipid indicators, provide important information regarding an individual’s physiological status and metabolic health. In contrast, genetic features, including gene variants and susceptibility loci associated with GDM, reflect inherited risk factors that may influence insulin secretion, insulin resistance, and glucose metabolism. Since clinical and genetic information provide complementary perspectives, integrating these heterogeneous data sources enables a more comprehensive characterization of GDM risk factors. The overall workflow of data preprocessing, feature fusion, and sample reconstruction is illustrated in Fig 1.

First, data cleaning was performed on the raw dataset, including outlier processing, missing value imputation, data type conversion, redundant data removal, and normalization, thereby ensuring data consistency and integrity. After data cleaning, the dataset was divided into training, validation, and test subsets at a ratio of 6:2:2. To avoid information leakage, all subsequent feature selection and parameter determination procedures were performed exclusively on the training set. Let the cleaned feature matrix and corresponding labels be represented by Eq 1 and Eq 2, respectively, where *N* denotes the number of samples and *d* denotes the number of features.


$$\begin{array}{c} \text{X}=[x_{\text{1}},x_{\text{2}},...,x_{N}{]}^{T}\in R^{N\times d} \end{array}$$ (1)

$$\begin{array}{c} Y=[y_{\text{1}},y_{\text{2}},...,y_{N}{]}^{T}\in R^{N} \end{array}$$ (2)

Feature selection algorithms were subsequently applied to the training set to evaluate the correlation between individual clinical and genetic features and GDM status. Based on the obtained relevance scores, the most informative features were retained for subsequent fusion. To further enhance feature representation and capture the interaction between genetic susceptibility and clinical factors, a correlation-driven weighted fusion method was adopted. Specifically, the top-k selected clinical and genetic features were integrated to construct a fused feature termed CSF. The construction of CSF is defined in Eq 3, where *cf*_*i*_ and *sf*_*i*_ denote the selected clinical and genetic features, respectively. The Scaling function normalizes clinical features into the range [0,1], while α and β represent the weights assigned to clinical and genetic information. The selected feature subset and the corresponding fusion strategy determined from the training set were subsequently applied to the validation and test sets to ensure consistency across all data subsets.


$$\begin{array}{c} CSF=\sum_{i=1}^{k}(\alpha \cdot Scaling(log(cf_{i}+1))+\beta \cdot sf_{i}) \end{array}$$ (3)

Moreover, accurately uncovering potential feature differences among subjects is important for enhancing the representation of population-level heterogeneity and individual risk characteristics. After feature fusion, sample reconstruction was performed independently on the training, validation, and test sets using a sliding-window mechanism. This mechanism transforms isolated individual features into context-aware group features. Specifically, for each sample sequence, a sliding window with a fixed length *L* and stride *T* was applied to generate reconstructed instances. The *i*-th reconstructed sample is defined by Eq 4 and Eq 5.


$$\begin{array}{c} {\text{X}}^{\left(i\right)}{\text{=[x}}_{i}{\text{,x}}_{i+1}{\text{,...,x}}_{i+L-1}\text{]}\in {\text{R}}^{L\times d} \end{array}$$ (4)

$$\begin{array}{c} Y^{\left(i\right)}=\left\{ \begin{array}{c} @l@y_{i}\;\;\;\;\text{if }T\text{=1}\\ {}[y_{i},y_{i+1},...,y_{i+T-1}]\text{ if }T\text{>1} \end{array}\right.\;\in {\text{R}}^{T}\; \end{array}$$ (5)

This reconstruction process generates datasets enriched with cross-individual contrastive features, thereby enhancing the model’s ability to capture complex feature interactions and latent subpopulation differences. The final reconstructed dataset is represented by Eq 6.


$$\begin{array}{c} D_{X}=\{X^{\left(i\right)}{\}}_{i=0}^{N-L-T},\;D_{Y}=\{Y^{\left(i\right)}{\}}_{i=0}^{N-L-T} \end{array}$$ (6)

To support efficient batch training and parallel processing, the reconstructed datasets were further converted into iterable DataLoader structures. Considering the potential class imbalance between high-risk and low-risk samples, direct random sampling may bias the model toward the majority class and reduce its sensitivity to minority-class instances. To address this issue, a WeightedRandomSampler was applied exclusively to the training set. Formally, let the training dataset contain *N* samples divided into *C* classes, where *n*_*i*_ denotes the number of samples belonging to class *i*. The sampling probability assigned to each sample in class *i* is defined by Eq 7.


$$\begin{array}{c} P_{i}=\frac{\text{1}}{C\cdot n_{i}} \end{array}$$ (7)

The validation and test sets retained their original sample distributions to ensure unbiased performance evaluation. This sampling strategy allows all classes to achieve approximately equal expected sampling frequencies during training, thereby mitigating class imbalance and improving the model’s ability to identify high-risk GDM cases.

### 3.2 Construction of the MT-GDM model

#### 3.2.1 The overall architecture of MT-GDM.

**Fig 2:**
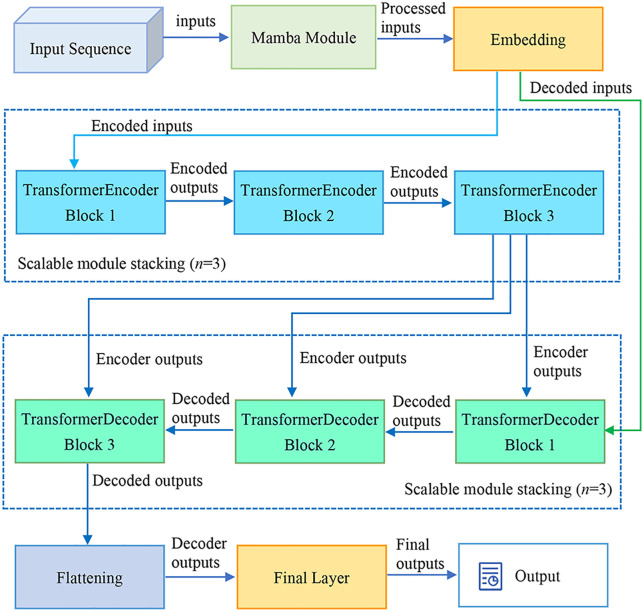
The overall architecture of the MT-GDM model.

To fully explore the complex interactions between clinical and genetic features of GDM, we propose the MT-GDM model, a novel hybrid architecture that combines the Mamba state space model with the Transformer framework. It can enhance the ability to conduct comparative analysis across sample sequences and improve the perception of individual heterogeneity. The overall architecture of MT-GDM is shown in Fig 2.

At the core of MT-GDM is a Transformer framework enhanced with embedded Mamba modules. This design leverages efficient information extraction and transmission mechanisms to improve prediction accuracy and generalization. The model operates as follows: First, the input data is processed by the Mamba module to extract initial sequence features. It can effectively capture global patterns, making it especially suitable for representing complex clinical and genetic data. The first output tensor of Mamba is passed through a linear embedding layer, where clinical and genetic features are mapped into a unified high-dimensional space. Next, the embedded features are input into both the Transformer encoder and decoder. This structure helps retain key input information. The main body of the model adopts a scalable multi-layer Transformer encoders and decoders. The encoder comprises multiple TransformerEncoder blocks to model the embedded features hierarchically. The decoder part receives the encoded inputs and recursively models the prediction target through the TransformerDecoder module. Both TransformerEncoder and TransformerDecoder use residual connections, multi-head attention mechanisms, and feedforward fully connected networks. These elements strengthen the model’s representational capacity and training stability. Finally, the decoded outputs are flattened and passed through a fully connected layer (Final Layer) to generate the final prediction.

In this architecture, the Mamba module acts as a feature extractor before the Transformer, enhancing the expression of dependencies. The symmetric stacking of multi-layer encoders and decoders enables hierarchical abstraction and transformation of input features. This process gradually extracts feature representations from low to high levels, facilitating the modeling of complex patterns in the pathogenesis of GDM. Moreover, the modular and scalable architecture provides flexibility for adjusting model complexity according to different application requirements.

#### 3.2.2 Mamba module.

We incorporated the Mamba module into the input layer of the Transformer to enhance the modeling of long-range dependencies in GDM features. Mamba, a recently proposed state space model (SSM), achieves an effective balance between long-term dependency representation and computational efficiency [43]. Specifically, the model represents sequence dependencies through state space equations, mapping a function (x(t)∈R) to a hidden state ($h\left(t\right)\in R^{m\times n}$) and subsequently producing the output (y(t)∈R), where *m* is the size of the state dimension and *n* is the sequence length. This process can be represented by Eq 8.


$$\begin{array}{c} \left\{ \begin{array}{c} @l@h^{\text{'}}\left(t\right)=Ah\left(t\right)+Bx\left(t\right)\\ y\left(t\right)=Ch\left(t\right)\; \end{array}\right. \end{array}$$ (8)

Among them, $A\in R^{m\times m}$ represents the state matrix; $B\in R^{m\times n}$ and $C\in R^{n\times m}$ represent the projection parameters; $h^{\text{'}}\left(t\right)$ is the hidden state of the current input *x*(*t*); *h*(*t*) represents the hidden state at the previous time point. Traditional SSM can only process continuous data, whereas deep learning tasks encompass both continuous and discre*t*e inpu*t*s. To meet the requirements of deep learning, Mamba discretizes the data form. The continuous SSM is transformed into a discrete SSM using the zero-order hold technique. The processing can be expressed as Eq 9 and Eq 10, respectively, where *I* represents the identity matrix. After discretization, the mapping between functions is transformed into the mapping of sequences *x*_*t*_ and *y*_*t*_.


$$\begin{array}{c} \left\{ \begin{array}{c} @l@\overset{―}{A}=exp\left(\Delta A\right)\;\\ \overset{―}{B}=(\Delta A{)}^{-1}[exp\left(\Delta A\right)-I]\cdot \Delta B \end{array}\right. \end{array}$$ (9)

$$\begin{array}{c} \left\{ \begin{array}{c} @c@h_{t}=\overset{―}{A}h_{t-1}+\overset{―}{B}x_{t}\\ y_{t}=Ch_{t}\text{\textbackslash{}hspace\{0.33em}\;\;\;\} \end{array}\right. \end{array}$$ (10)

**Fig 3:**
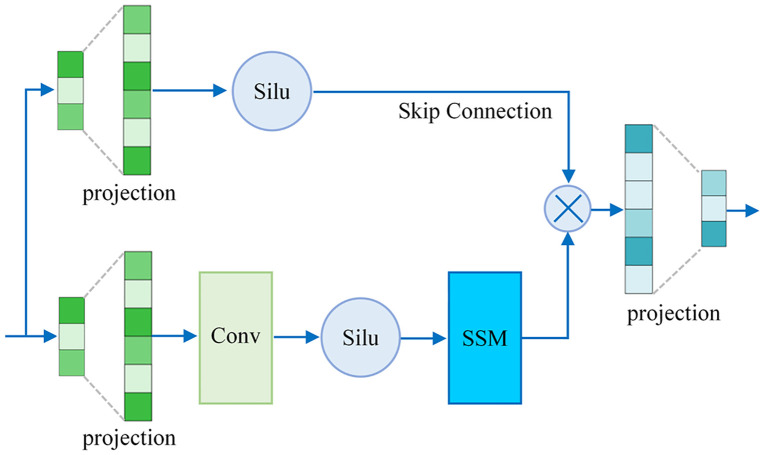
The structure of the Mamba module.


The structure of the Mamba module is shown in Fig 3. It has two parallel branches. In the first branch, the feature channels are linearly projected and then processed through a convolution operation. Then the feature transformation is achieved through the SiLU activation function and SSM. In the second branch, it is also linearly projected and activated by the SiLU function. Then, the features of the two branches are aggregated through the Hadamard product. Finally, the aggregation result is linearly projected to generate output. The processing flow can be summarized as Eq 11.

$$\begin{array}{c} \left\{ \begin{array}{c} @l@Branch_{\text{1}}=SSM\left(SiLU\left(Conv\left(Linear\left(input\right)\right)\right)\right)\\ Branch_{\text{2}}=SiLU\left(Linear\left(input\right)\right)\;\\ Branch_{out}=Linear\left(Branch_{\text{1}}⊙Branch_{\text{2}}\right) \end{array}\right. \end{array}$$ (11)

As a preprocessing unit for MT-GDM feature extraction, the Mamba module can effectively capture long-distance sequence dependencies. It is crucial for mining the changing trends of clinical data and the long-range interactions of gene loci in genetic data. The structural design of the Mamba module supports parallel computing and can quickly generate feature representations, providing timely data support for the subsequent Transformer module processing.

#### 3.2.3 Design of the transformerencoder module.

As the core component of the model, TransformerEncoder performs deep semantic modeling on the features preprocessed by the Mamba Module. It captures the complex interactions within the sequence through the multi-head self-attention mechanism and uses the feedforward network to transform and enhance the features. Multi-head attention executes multiple independent attention mechanisms in parallel to obtain the subspace attention distribution of the input sequence. In multi-head attention, the input sequence passes through three linear layers to obtain the query, key, and value, respectively. These transformed vectors are divided into several attention heads, each with its own independent query, key, and value matrices. All head outputs are concatenated together to obtain the final attention output vector. The output of a single attention head can be represented as Eq 12, where *Q*, *K*, and *V* represent query, key, and value matrices, respectively; *d*_*k*_ represents the ratio of feature dimension to the number of attention heads; softmax is used to convert similarity into a probability distribution.


$$\begin{array}{c} Attention\left(Q,K,V\right)=softmax\left(\frac{QK^{T}}{\sqrt{d_{k}}}\right)V \end{array}$$ (12)

**Fig 4:**
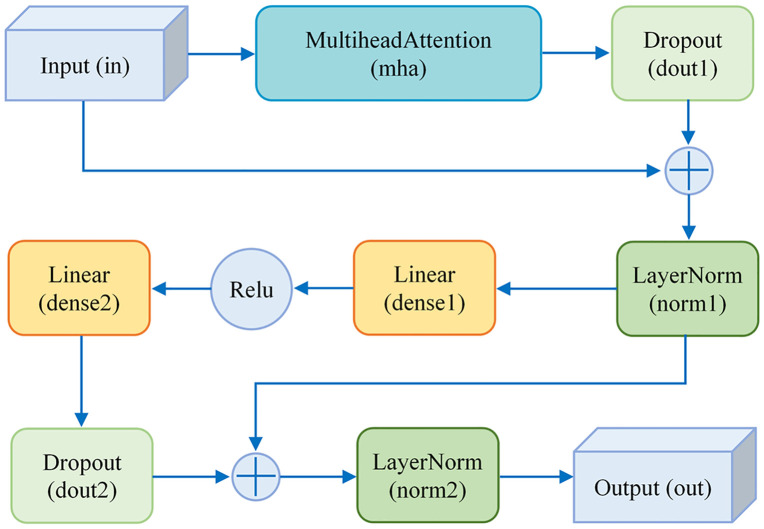
The structure of the TransformerEncoder module.

The TransformerEncoder we designed integrates key components such as multi-head self-attention, residual connection, and layer normalization. The structure of TransformerEncoder is shown in Fig 4.

The execution of TransformerEncoder starts with receiving a feature sequence preprocessed by the Mamba module and mapped to a specific dimension. The sequence first enters the multi-head self-attention module, where the attention output is processed by dropout and then combined with the original input through a residual connection. This result then passes through a layer normalization step to stabilize the data distribution, producing the intermediate feature. The intermediate feature is then passed through a feedforward network composed of two linear layers, followed by dropout for regularization. Finally, the output is residually connected with the intermediate feature of the previous stage again, and the final encoding representation is obtained by layer normalization.

The overall process leverages multi-head self-attention to capture global dependencies, while residual connections and layer normalization facilitate smooth information flow and stable training. In MT-GDM, multiple stacked TransformerEncoder blocks hierarchically abstract the input features. As the subsequent module to Mamba, they form a collaborative architecture that bridges efficient sequence modeling with deep semantic understanding, ultimately providing high-quality contextual representations for the decoder modules.

#### 3.2.4 Design of the transformerdecoder module.

**Fig 5:**
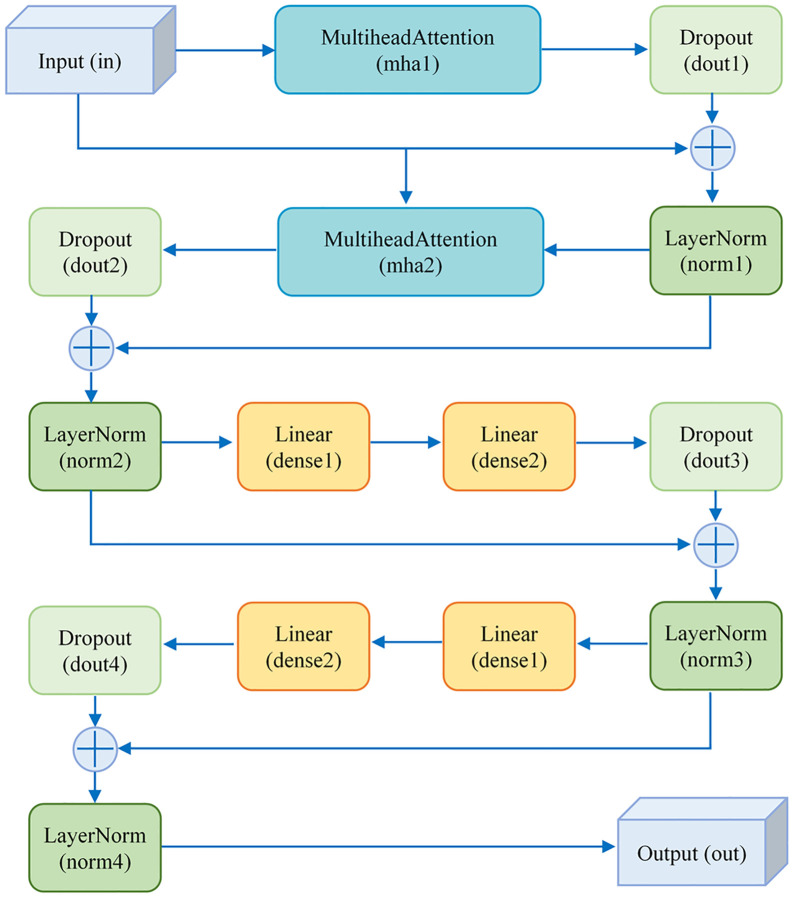
The structure of the TransformerDecoder module.

In the MT-GDM model, the TransformerDecoder module serves as a key component in the decoding process. It mainly converts the contextual information extracted by the encoder into an output sequence. Its core design uses a dual-attention mechanism and deep feature transformation. This allows the decoder to make full use of encoder outputs and to generate the target sequence dynamically. The design of TransformerDecoder is shown in Fig 5. It mainly includes self-attention output sequence modeling, encoder-decoder attention interaction fusion, and multi-layer feature transformation with efficient information transmission.

First, the input sequence passes through a multi-head self-attention layer to model dependencies within the sequence. Subsequent dropout regularization, residual connection, and layer normalization preserve the original information and improve training stability. Next, a second multi-head attention layer fuses the current decoding state with the encoder output, enabling global context integration from the input sequence. This process also incorporates residual connections and normalization to provide deep semantic representations for the output sequence. Finally, the fully connected layer is reused and combined with residual connection and layer normalization for feature conversion. This design alleviates the gradient vanishing problem of deep networks and achieves accurate mapping from context features to target outputs.

The entire process achieves multi-layer feature transformation and efficient transmission through a dual-attention mechanism, nested residual connections, and normalization. The final output is a decoder representation enriched with semantic information.

## 4 Experiments

This section presents the composition of the GDMRPD dataset, the encoding representation of genetic information, the experimental procedures, and the experimental results. The experiments were conducted on an Ubuntu 22.04 64-bit operating system, equipped with an Intel Xeon (Ice Lake) Platinum 8369B CPU, 64 GB of RAM, and an NVIDIA A10 GPU. The deep learning framework PyTorch was used for model implementation.

To comprehensively evaluate the model’s performance, multiple evaluation metrics are employed in GDM prediction. Specifically, these metrics include Sensitivity, Specificity, Positive Predictive Value (PPV), and Negative Predictive Value (NPV), and F1-score. Among these metrics, higher Sensitivity reflects the model’s ability to identify individuals at potential risk of GDM, while higher Specificity reduces the likelihood of misjudging healthy individuals and unnecessary medical interventions. A higher PPV indicates the reliability of positive predictions, whereas a higher NPV demonstrates the effectiveness of correctly excluding low-risk cases. The F1-score, as the harmonic mean of Sensitivity and PPV, provides a balanced evaluation of overall performance. In addition, we also analyzed the AUC metric, which reflects the overall discriminative ability of the model. In practice, an appropriate threshold must be set to convert predicted probabilities into class labels. The threshold directly affects the balance between Sensitivity and Specificity, thereby significantly influencing model performance under different application scenarios.

### 4.1 The DMRPD dataset

**Fig 6:**
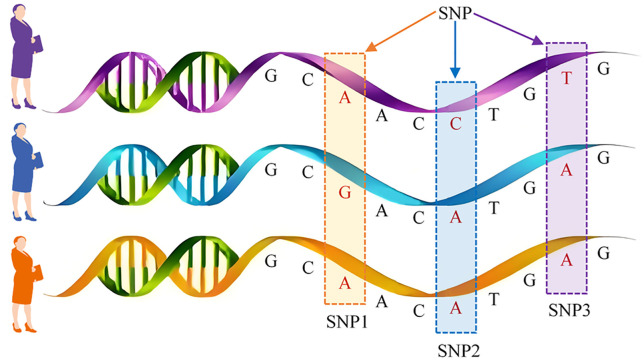
The feature representation of genetic information.

The DMRPD dataset contains extensive clinical features along with selected genetic variant markers. Each record includes 28 clinical attributes, such as age, parity, blood glucose levels, BMI, blood pressure, blood lipid profiles, and family history of diabetes, as well as 55 genetic features, including genotype information. In addition, the dataset provides an ID feature and a disease status label. Since the ID feature functions only as a sample identifier, it was excluded from the analysis. Clinical features are presented as numerical or categorical variables, while genetic features are encoded using a three-state scheme (1/2/3) to denote the genotype of a Single Nucleotide Polymorphism (SNP) locus. An SNP is a variation at a single position in the DNA sequence among individuals and is widely used as a genetic marker in genome-wide studies. The genotypes of SNPs are encoded using the numbers 1, 2, and 3 to represent different allele combinations at a specific locus. The representation of genetic features is shown in Fig 6, which indicates the genotypes of three SNP loci. These genetic features do not directly correspond to nucleotide bases but are numerically encoded to facilitate analysis. The meanings of the SNP values are summarized in Table 1.

**Table 1:** The meaning of SNP values.

Code	Biological meaning	Example (Alleles A and G)
1	Homozygous for the reference allele	AA (reference allele is A)
2	Heterozygous genotype	AG or GA
3	Homozygous for the variant allele	GG (variant allele is G)

The reference allele refers to the allele that is most common in the population or specified in the reference genome at a specific genetic locus. It is typically regarded as the “standard” or “normal” version and serves as a baseline for comparing the alleles found in other individuals. In contrast, the variant allele is the nucleotide base that differs from the reference allele at that same locus. It usually reflects a mutation or genetic variation in an individual, which may affect gene function or be neutral in effect. This distinction between reference and variant alleles is fundamental to SNP-based genetic analysis.

### 4.2 Experiment procedures

#### 4.2.1 Data cleaning.

Initially, both clinical and genetic features in the DMRPD dataset undergo preprocessing. Missing values are imputed using filling strategies, and outliers are identified and removed to ensure a reasonable data distribution. Numerical features are normalized, categorical variables are encoded, and irrelevant or redundant features are eliminated to improve computational efficiency.

For the features “Gravidity” and “Parity,” missing values were imputed using the median. Additionally, outliers where “Parity” exceeded “Gravidity” were corrected by setting “Parity” equal to “Gravidity”. Considering biological correlations, the missing values of “SBP”, “DBP”, and “ScreenWeek” were imputed using the group-wise mean based on “Age”, “Gravidity”, and “Parity”. This approach preserves the intra-group distribution more effectively than global averaging. Other numerical clinical features were imputed using the overall mean. For missing values in certain SNP loci and categorical clinical features, a default value of 0 was used to represent an unknown state. Since the “PreBMI” value is directly related to “Height” and “Weight”, the “Height” and “Weight” features were removed to avoid redundancy. In addition, clinical features with more than 50% missing values were excluded from the dataset. The “ID” feature, being merely a sample identifier, was also discarded. After these preprocessing steps, all numerical features are normalized, and categorical features are encoded using TargetEncoder, ensuring data integrity and consistency for subsequent model training.

#### 4.2.2 Feature selection and fusion.

**Fig 7:**
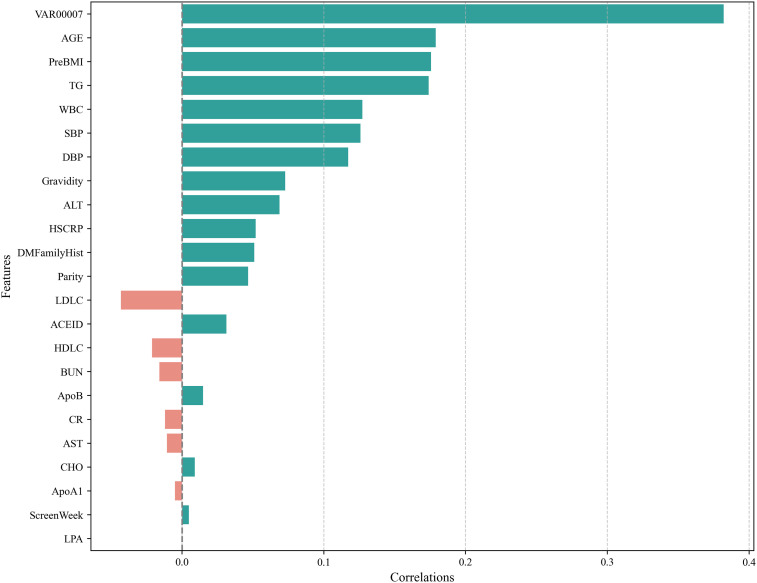
The correlation between clinical features and GDM status.

**Fig 8:**
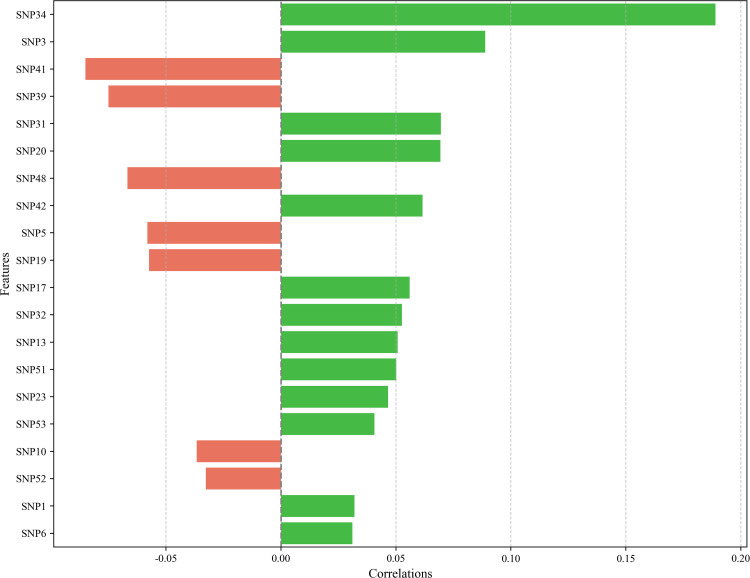
The correlation between genetic features and GDM status (top 20).

In the feature engineering stage, multiple techniques were applied to select and integrate both clinical and genetic features in order to improve the predictive performance of the proposed model. To prevent potential data leakage, all feature selection, correlation analysis, and feature ranking procedures were performed exclusively on the training set. First, the Pearson correlation coefficient was employed to evaluate the linear relationships between clinical features and GDM status, as illustrated in Fig 7. A similar analysis was performed for genetic features (SNP loci), and the top 20 SNPs showing the strongest correlations with GDM status were identified, as shown in Fig 8. Based on these results, clinical and genetic features with weak or negligible correlations were removed during feature fusion to reduce feature redundancy and potential noise.

Next, to further enhance feature representation, a fused feature termed CSF was constructed using a correlation-driven weighted fusion strategy, as defined in Eq 3. Specifically, the top-k clinical features and the top-k genetic features ranked by Pearson correlation coefficients were selected for feature fusion. Different values of k were evaluated by training the model on the training set and assessing its performance on the validation set. The corresponding validation AUC results are summarized in Table 2.

**Table 2:** Validation performance under different values of k used for feature fusion.

k Value	Number of features used for fusion	Validation AUC
1	2	0.824
2	4	0.835
3	6	0.847
4	8	0.841
5	10	0.838
6	12	0.831
8	16	0.829
10	20	0.821

As shown in Table 2, model performance improved as the number of selected features increased from k = 1 to k = 3, peaking at k = 3. Beyond this point, adding more features yielded no further gains. In some cases, validation performance slightly decreased. This suggests that lower-ranked features introduced redundant or less informative signals. Consequently, the top three clinical and genetic features were selected for fusion. This choice provides a favorable balance between predictive performance, model stability, and interpretability while preserving the most informative signals from both clinical and genetic sources.

The weighting coefficients (α and β) in Eq 3 were introduced to balance the contributions of clinical and genetic information in the fused representation. In this study, α and β were set to 0.5 to provide equal contributions from clinical and genetic features. This setting prevents either feature group from dominating the fusion process and enables the fused feature to capture complementary effects between clinical risk factors and genetic susceptibility.

**Fig 9:**
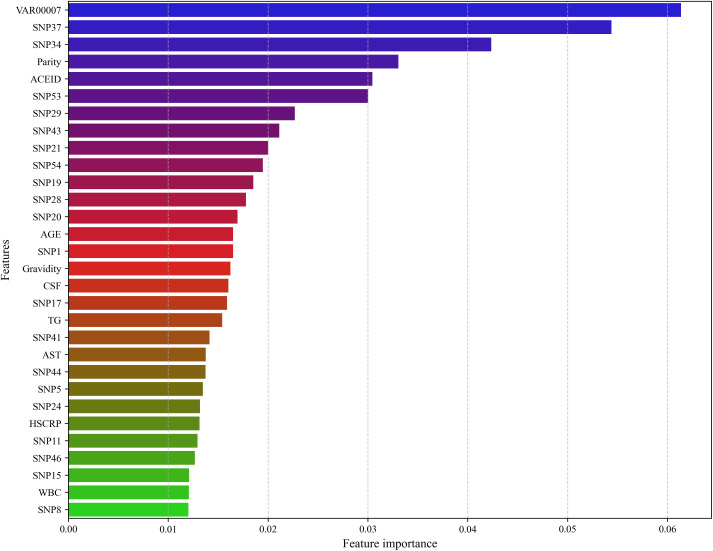
The importance scores after feature fusion (top 30).

Finally, a Random Forest model was employed to evaluate the importance of all input features. Fig 9 presents the features with importance scores ranked within the top 30%. Notably, the CSF feature is included among these highly ranked features, indicating that the proposed fusion strategy provides meaningful predictive information. This finding suggests that the proposed feature fusion strategy enhances the informativeness of the feature space and highlights its practical value.

#### 4.2.3 Sample reconstruction.

After data preprocessing and feature fusion, the window length *L* and stride *T* are defined by Eq 4 and Eq 5. These parameters are used to segment sequential instances and generate training sequences that capture comparative features and contextual dependencies. Sample reconstruction was performed separately on the training, validation, and testing sets. To facilitate efficient batch training and parallel processing, the reconstructed datasets were converted into iterable DataLoader structures. In summary, the reconstructed samples provide augmented instances for model input, enabling better representation of population-level heterogeneity and individual risk.

#### 4.2.4 Model training and comparison setup.

In this study, model training and comparison setup were designed to evaluate the performance of the proposed MT-GDM model. To provide a comprehensive assessment, MT-GDM was compared with several representative models commonly used for risk prediction tasks. The MT-GDM model integrates Mamba and Transformer components, combining the strengths of long-range dependency modeling and deep feature representation learning. To enhance training robustness, PyTorch’s WeightedRandomSampler was employed to achieve balanced sampling across labels, thereby improving fair representation of minority classes during training. All models were trained and evaluated using the same training, validation, and test datasets under identical experimental settings to ensure a fair comparison. The main training configurations used in this study are summarized in Table 3.

**Table 3:** Training configurations for MT-GDM and baseline models.

Parameter	Parameter value
Window length (L)	4
Stride (T)	1
Embedding dimension	16
Number of attention heads	4
Feed-forward dimension	16
Activation function	Sigmoid
Random seed	42
Optimizer	Adam
Learning rate	0.001
Batch size	32
Epochs	100
Dropout rate	0.1
Loss function	BCELoss

The performance of MT-GDM was compared with the following representative models:

**Mamba:** A state-of-the-art linear state-space model, used here as a standalone predictor to assess its effectiveness in long-sequence modeling.

**Transformer:** A standard Transformer architecture without the Mamba module, serving as a baseline for comparison with hybrid structures.

**LightGBM:** An efficient gradient boosting framework, widely used for structured data tasks in the medical domain.

**CatBoost:** A gradient boosting model particularly effective in handling categorical variables and heterogeneous feature spaces.

These comparison settings establish a consistent experimental framework for evaluating the predictive performance of the proposed MT-GDM model.

### 4.3. Experimental results

**Fig 10:**
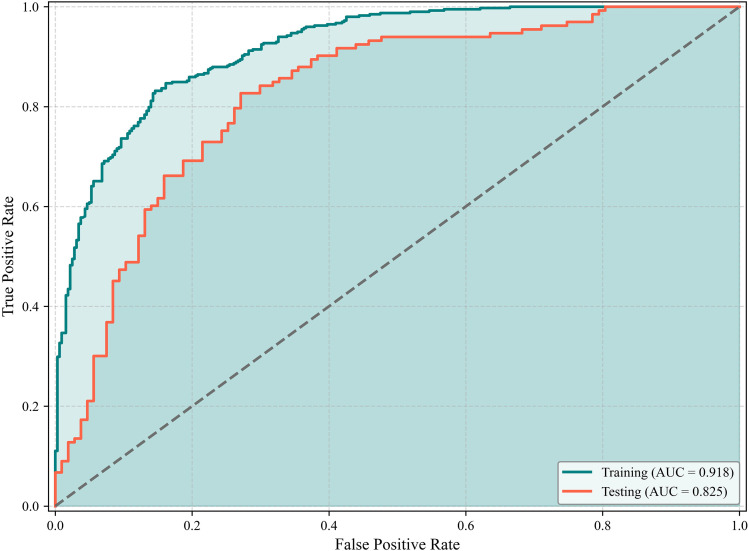
The ROC curves of MT-GDM on the training and test sets.

**Fig 11:**
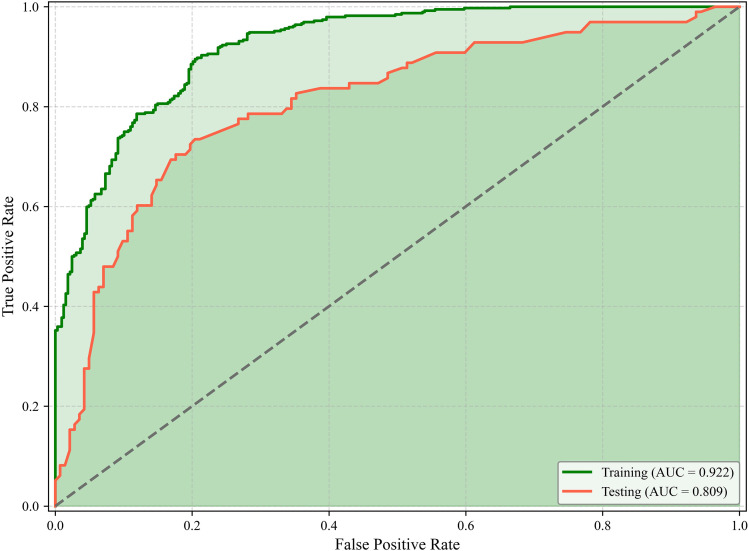
The ROC curves of Transformer on the training and test sets.

**Fig 12:**
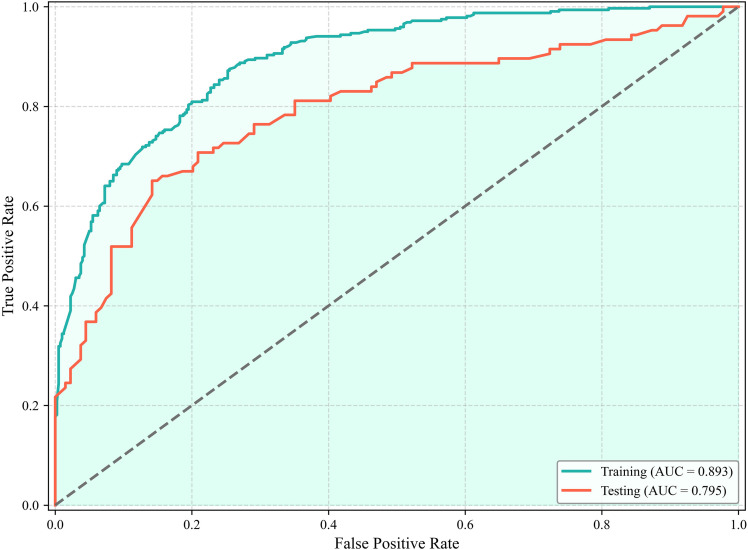
The ROC curves of Mamba on the training and test sets.

In this study, we used the PyTorch framework to construct the MT-GDM model. We also encapsulated and defined the Mamba, Transformer, LightGBM, and CatBoost classes for comparative experiments. All models were trained for 100 epochs, and the best-performing model on the validation set was selected for evaluation on the test set. The ROC curves of MT-GDM on both the training and test sets are shown in Fig 10. The model achieved an AUC of 0.918 on the training set and 0.825 on the test set, indicating high predictive accuracy while maintaining robust generalization capability. Fig 11 presents the ROC curves for the Transformer model, a variant of MT-GDM that removes the Mamba module. Although the Transformer achieved a slightly higher AUC on the training set (0.922), its test-set AUC decreased to 0.809, whereas MT-GDM achieved a higher AUC on the test set (0.825), suggesting improved generalization performance of the proposed model. Fig 12 presents the ROC curves for the Mamba model, which only contains the Mamba module, followed by fully connected layers. The AUC values for the training and test sets were 0.893 and 0.795, respectively, demonstrating that Mamba alone is effective but lacks the deep representation capacity of MT-GDM.

**Fig 13:**
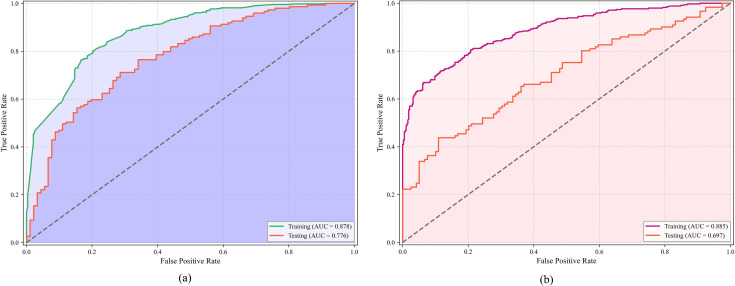
(a) The ROC curves of LightGBM on the training and test sets. (b) The ROC curves of CatBoost on the training and test sets.

In addition, LightGBM and CatBoost have significant advantages in structured tasks or heterogeneous feature processing. As models widely used in medical applications, they are also included in the comparative experiments. Fig 13 displays the ROC curves of LightGBM and CatBoost, respectively. LightGBM achieved AUCs of 0.878 and 0.776 on the training and test sets, respectively. CatBoost performed slightly better on the training set (AUC = 0.885) but significantly worse on the test set (AUC = 0.697), indicating overfitting and lower generalization.

**Fig 14:**
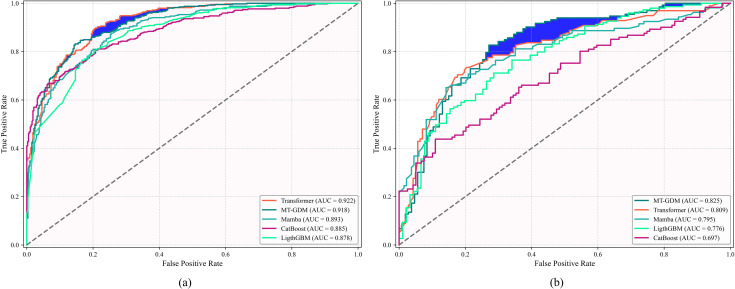
(a) The comparison of ROC curves of five models on the training set. (b) The comparison of ROC curves of five models on the test set.

To more clearly compare the overall performance of the five models, we plotted their ROC curves on both the training and test sets. Fig 14 presents the ROC curves of all models on both datasets. The blue-shaded area highlights the model achieving the highest AUC in each dataset. As shown in Fig 14 (a), the Transformer achieved the highest AUC on the training set, whereas MT-GDM achieved the highest AUC on the test set in Fig 14 (b), suggesting better generalization capability of the proposed model on this dataset. To further assess the statistical uncertainty of model performance, 95% confidence intervals (CI) for the AUC values were estimated using 1000 bootstrap resamples on the test set. MT-GDM achieved the highest AUC of 0.825 (95% CI: 0.770–0.877) among all evaluated models, indicating stable predictive performance. Although the confidence intervals of several models partially overlap, MT-GDM consistently maintained superior or competitive performance across multiple evaluation metrics. Detailed performance metrics of each model on both the training and test sets, including AUC (95% CI), optimal threshold, Sensitivity, Specificity, PPV, NPV, and F1-score, are summarized in Table 4.

**Table 4:** The performance of five models on training and test sets.

Dataset	Model	AUC (95% CI)	Threshold	Sensitivity	Specificity	PPV	NPV	F1
Training set	MT-GDM	0.918 (-)	0.555	0.832	0.854	0.876	0.804	0.853
	Transformer	0.922 (-)	0.503	0.895	0.796	0.840	0.864	0.867
	Mamba	0.893 (-)	0.435	0.875	0.745	0.733	0.882	0.798
	LightGBM	0.878 (-)	0.611	0.802	0.797	0.857	0.726	0.829
	CatBoost	0.885 (-)	0.547	0.669	0.936	0.906	0.755	0.770
Test set	MT-GDM	0.825 (0.770-0.877)	0.526	0.827	0.729	0.791	0.772	0.809
	Transformer	0.809 (0.747-0.865)	0.527	0.735	0.796	0.713	0.813	0.724
	Mamba	0.795 (0.734-0.851)	0.499	0.651	0.858	0.784	0.757	0.711
	LightGBM	0.776 (0.713-0.834)	0.623	0.711	0.714	0.803	0.602	0.754
	CatBoost	0.697 (0.627-0.762)	0.570	0.438	0.891	0.803	0.609	0.567

Furthermore, pairwise comparisons of AUC values between MT-GDM and the baseline models were conducted using the DeLong test, as presented in Table 5. The results indicate that MT-GDM achieved a statistically significant improvement over CatBoost (p = 0.004). In contrast, the differences between MT-GDM and the Transformer, Mamba, and LightGBM models did not reach statistical significance at the 0.05 level. Nevertheless, MT-GDM achieved the highest observed AUC on the test set and demonstrated competitive performance across multiple evaluation metrics, including Sensitivity, PPV, and F1-score. These findings suggest that MT-GDM provides reliable predictive performance for GDM risk prediction.

**Table 5:** Pairwise DeLong test for AUC comparison on the test set.

Model Comparison	AUC Difference	p-value
MT-GDM vs Transformer	0.016	0.214
MT-GDM vs Mamba	0.030	0.129
MT-GDM vs LightGBM	0.049	0.061
MT-GDM vs CatBoost	0.128	0.004

Overall, the experimental results on the test set demonstrate that MT-GDM achieves well-balanced performance across multiple evaluation metrics. The model obtained a Sensitivity of 0.827 and a Specificity of 0.729, suggesting effective identification of both GDM cases and non-GDM individuals. The PPV (0.791) and NPV (0.772) further indicate reliable predictive performance for both positive and negative classifications. In terms of the F1-score, MT-GDM achieved 0.809, outperforming all other comparison models. In addition, bootstrap-estimated 95% confidence intervals for AUC and DeLong tests were conducted to assess the statistical uncertainty of model performance. Taken together, these findings suggest that MT-GDM represents a competitive approach for GDM risk prediction, with performance evaluated through confidence intervals and statistical comparisons.

## 5 Discussion

The experimental results demonstrate that the proposed MT-GDM model achieves superior predictive performance on the test set compared with four representative comparison models. These findings suggest that the proposed method may help alleviate some limitations of existing approaches. From a methodological perspective, the improvements of MT-GDM can largely be attributed to three main aspects. First, the combination of feature fusion and sample reconstruction provides a more comprehensive representation of potential GDM risk, which is consistent with recent studies emphasizing the importance of multi-source feature integration and data augmentation in disease prediction. Second, the Mamba module placed before the Transformer encoder enables more effective capture of long-range dependencies in sequences, thereby enhancing the recognition of complex patterns. Compared with the standalone Transformer model, the superior test-set performance of MT-GDM suggests that the Mamba module may provide complementary sequence modeling capabilities and help improve generalization by reducing reliance on dataset-specific patterns. Third, the flexible and extensible architecture of MT-GDM facilitates structural adaptation and future model enhancement, allowing additional modules or feature representations to be incorporated with minimal modification to the overall framework.

Beyond the architectural advantages of the model, examining the contributions of individual features provides further insight into the predictive mechanism of MT-GDM. As shown in Fig 9, the fused CSF feature constructed from multiple clinical features (VAR00007, AGE, and PreBMI) play an important role in the prediction process. Among these features, maternal age and pre-pregnancy BMI are well-established risk factors for GDM and are closely associated with metabolic status during pregnancy [44]. Although the clinical meaning of the feature VAR00007 is not explicitly annotated in the dataset, its relatively high importance score suggests that it may capture additional information related to GDM risk. Furthermore, the CSF feature integrates clinical indicators with genetic susceptibility information, enabling the model to exploit complementary relationships between these heterogeneous data sources. From a clinical perspective, such integration of heterogeneous health data may provide complementary information for GDM risk prediction. It may also help improve risk stratification and enable earlier identification of pregnant women at high risk of developing GDM.

Nevertheless, this study has several limitations. The DMRPD dataset used in this study is limited in both size and scope, which may affect the generalizability of the findings to other populations and clinical settings. In addition, only a limited number of SNP loci were included in this study. Expanding the genomic data could potentially enhance the predictive capacity of the model. Future studies incorporating larger multi-center cohorts and more comprehensive genomic features may further strengthen the robustness and applicability of the model.

## 6 Conclusion

In this paper, we propose the MT-GDM model that combines the strengths of Mamba and Transformer architectures. The correlation-driven weighted fusion strategy and the sample reconstruction mechanism provide a more comprehensive representation of GDM-related risk factors. In addition, the modular and extensible design of the framework enhances its flexibility and scalability for future model development.

Experimental evaluations on the DMRPD dataset indicate that MT-GDM achieved competitive performance compared with several representative models, including LightGBM, CatBoost, Mamba, and the standard Transformer. On the independent test set, MT-GDM achieved an AUC of 0.825 while maintaining balanced Sensitivity and Specificity, suggesting its potential utility for identifying individuals at different levels of GDM risk.

Overall, MT-GDM represents a promising approach for GDM risk prediction based on the integration of clinical and genetic information. However, given that the current study was conducted using a single dataset, further validation on larger, more diverse, and independent cohorts is necessary to confirm its generalizability and potential clinical applicability. Future work will focus on incorporating longitudinal clinical data and more comprehensive genomic features to further evaluate and enhance the robustness of the proposed framework.

## Supporting information

S1 Data Anonymized clinical and genetic dataset used in this study. (ZIP)
