# Supplementary material for: A hybrid mamba-transformer architecture fusing clinical and genetic features for gestational diabetes mellitus prediction
Source: PLoS One. 2026 Aug 12;21(8):e0355729. doi: 10.1371/journal.pone.0355729 (PMC13465813; doi:10.1371/journal.pone.0355729)
Supplement: Supplementary file 1 [file pone.0355729.s001.zip › description.docx]

**The raw data comes from the following references:**

Alibaba Cloud Tianchi. Tianchi Precision Medicine Competition: AI-assisted Diabetes Mellitus Risk Prediction Dataset (DMRPD). Alibaba Cloud Tianchi Competition Platform; 2018 [cited 15 August 2025]. Available from: https://tianchi.aliyun.com/competition/entrance/231638/information

**The Chinese titles in the raw data have been translated into English, and some samples with missing labels have been removed. All data involving potential privacy concerns have been fully anonymized to ensure that no personally identifiable information is included. The meaning of each column in the dataset is as follows:**

Column 1: Id

Column 2: Retinol binding protein 4 (RBP4)

Column 3: Age

Column 4: Gravidity

Column 5: Parity

Column 6: Height

Column 7: Pre pregnancy weight

Column 8: BMI classification

Column 9: Pre pregnancy BMI

Column 10: Systolic blood pressure

Column 11: Diastolic pressure

Column 12: Blood pressure monitored during childbirth

Column 13: Down syndrome screening during pregnancy

Column 14: White Blood Cell (wbc)

Column 15: Clinical features identified as VAR00007

Column 16: Alanine Aminotransferase (ALT)

Column 17: Aspartate transaminase (AST)

Column 18: Serum creatinine (Cr)

Column 19: Blood Urea Nitrogen (BUN)

Column 20: Cholesterol (CHO)

Column 21:Thyroglobulin (TG)

Column 22: High-Density Lipoprotein Cholesterol (HDLC)

Column 23: Low Density Lipoprotein Cholesterol (LDLC)

Column 24: Apolipoprotein A1 (ApoA1)

Column 25: Apolipoprotein B (ApoB)

Column 26: Lipoprotein a (Lpa)

Column 27: High Sensitivity c Reactive Protein (HSCRP)

Column 28: DM Family History

Column 29: Clinical features identified as ACEID

Column 30-84: Single nucleotide polymorphism (SNP) loci SNP1-SNP55

Column 85: label
